# The epithelial-to-mesenchymal transition induced by tumor-associated macrophages confers chemoresistance in peritoneally disseminated pancreatic cancer

**DOI:** 10.1186/s13046-018-0981-2

**Published:** 2018-12-11

**Authors:** Kazuya Kuwada, Shunsuke Kagawa, Ryuichi Yoshida, Shuichi Sakamoto, Atene Ito, Megumi Watanabe, Takeshi Ieda, Shinji Kuroda, Satoru Kikuchi, Hiroshi Tazawa, Toshiyoshi Fujiwara

**Affiliations:** 1 Department of Gastroenterological Surgery, Okayama University Graduate School of Medicine, Dentistry and Pharmaceutical Sciences, 2-5-1 Shikata-cho, Kita-ku, Okayama, 700-8558 Japan; 2 Minimally Invasive Therapy Center, Okayama University Hospital, Okayama, Japan; 3 Center for Innovative Clinical Medicine, Okayama University Hospital, Okayama, Japan

**Keywords:** Tumor-associated macrophages, Chemoresistance, Epithelial-to-mesenchymal transition, Peritoneal dissemination, Pancreatic cancer

## Abstract

**Background:**

The peritoneum is one of the most frequent metastatic sites in pancreatic cancer patients, and peritoneal dissemination makes this disease refractory due to aggressive progression and chemoresistance. Although the role of the tumor microenvironment in cancer development is recognized, the correlation between the peritoneal environment and refractoriness of peritoneal dissemination remains unclear. The intraperitoneal tumor-microenvironment and its potential role in the progression of peritoneal dissemination and chemo-refractoriness, focusing especially on macrophages, were investigated.

**Materials and methods:**

Peritoneal washes were obtained from pancreatic cancer patients, and cellular components were subjected to immunofluorescence assays. The effects of macrophages induced from monocytic THP-1 cells on pancreatic cancer cells were examined in co-culture conditions. The in vivo effects of macrophages on tumor growth and chemo-sensitivity were investigated by subcutaneously or intraperitoneally co-injecting cancer cells with macrophages into mice.

**Results:**

CD204-positive macrophages were present along with cancer cells in the peritoneal washes. In in vitro co-culture, tumor-associated macrophages affected pancreatic cancer cells, induced the epithelial-to-mesenchymal transition (EMT), and made them more resistant to chemotherapeutic agents. M2 macrophages promoted growth of both subcutaneous tumors and peritoneal dissemination in mice. Furthermore, co-inoculation of M2 macrophages conferred chemoresistance in the peritoneal dissemination mouse model, which significantly shortened their survival.

**Conclusion:**

Intraperitoneal tumor-associated macrophages potentially play an important role in promotion of peritoneal dissemination and chemoresistance of pancreatic cancer via EMT induction.

**Electronic supplementary material:**

The online version of this article (10.1186/s13046-018-0981-2) contains supplementary material, which is available to authorized users.

## Background

Pancreatic cancer is the fifth most common cause of cancer-related deaths in the world [1, 2], and the prognosis of patients with pancreatic cancer is extremely poor even in resected cases, because pancreatic cancer frequently recurs [3]. The peritoneum is the second most common site of recurrence [4, 5], and patients with peritoneal dissemination are refractory to chemotherapy [6, 7]. However, the underlying mechanism leading to peritoneal dissemination and its refractoriness remains unclear. Thus, elucidation of the mechanism of peritoneal dissemination in pancreatic cancer and the development of effective strategies to treat it are urgently needed.

From tumor initiation to progression, the interactions between cancer cells and their surrounding tumor microenvironment (TME) play important roles [8–11]. The TME of solid tumors consists of various kinds of cells, including endothelial cells, fibroblasts, lymphocytes, and macrophages, and it has been vigorously investigated even in pancreatic cancer [12]. Whereas the peritoneal cavity is a large free space in the human body and contains sparse amounts of various types of cells, the intraperitoneal TME (ipTME) has been ignored and not well explored. In this study, the ipTME was investigated, focusing especially on the effects of tumor-associated macrophages on pancreatic cancer cells in the process of peritoneal dissemination.

## Methods

### Cell lines and cell cultures

Human pancreatic cancer cell lines (Panc1, BxPC-3) and a human acute monocytic leukemia cell line (THP-1) were purchased from the American Type Culture Collection (Manassas, VA, USA). Luciferase-expressing BxPC-3 cells (BxPC-3-luc) were purchased from the Japanese Collection of Research Bioresources Cell Bank (Osaka, Japan). Panc1 cells were cultured in DMEM (Sigma-Aldrich, St. Louis, MO, USA) supplemented with 10% FBS and 1% penicillin/streptomycin (Sigma-Aldrich). Other cell lines were cultured in RPMI1640 (Sigma-Aldrich) supplemented with 10% FBS and 1% penicillin/streptomycin. Cells were maintained at 37 °C in a humidified incubator in an atmosphere of 95% air and 5% CO_2_.

### Clinical sample preparation and cancer cell imaging by TelomeScan

Peritoneal washes were obtained from 34 pancreatic cancer patients during surgery. The peritoneal cavity was washed with 100 ml of normal saline, and the washes were collected from the peritoneal cavity near the pancreas. Half of each collected sample was subjected to pathological cytology examination, and the other half was used for the study. The institutional review board of Okayama University Graduate School approved the study protocol, and informed consent was obtained from all patients. All procedures were in accordance with the Helsinki Declaration.

TelomeScan is a genetically engineered adenovirus that replicates and expresses GFP only in telomerase-active cancer cells [13, 14]. The clinical peritoneal washes were centrifuged, and the cell pellets were resuspended in RPMI-1640. The cells were infected with TelomeScan for 24 h at 1 multiplicity of infection (MOI) according to the total number of viable cells. The GFP-expressing cells were recognized as cancer cells.

### Immunofluorescence staining

The cells were stained with anti-CD45 mouse IgG1 (HI30; BioLegend, San Diego, CA, USA), PE-conjugated anti-CD14 mouse IgG2a (M5E2; BioLegend), anti-CD204 mouse IgG1 (MSR-A; TransGenic, Fukuoka, Japan) or PE-conjugated anti-CD204 mouse IgG1 (REA148; Miltenyi Biotec, Bergisch Gladbach, Germany). As secondary antibodies, Alexafluor647-goat anti-mouse IgG (Invitrogen; Life Technologies Corporation; Carlsbad, CA, USA) was used. After immunofluorescence staining, the samples were observed under an inverted fluorescence microscope (IX71; Olympus, Tokyo, Japan).

### Induction of THP-1 monocytes to macrophages

The macrophage-like state was obtained by treating THP-1 monocytes for 48 h with 100 ng/ml phorbol 12-myristate 13-acetate (PMA; Sigma-Aldrich) in 24-well cell culture plates with 0.5 ml of cell suspension (5 × 10^5^cells) in each well. Differentiated, adherent cells were washed twice with culture medium (RPMI 1640 medium without PMA but containing 10% FBS and 1% penicillin/streptomycin) and rested for another 24 h in the culture medium to obtain the resting state of macrophages (M0). M0 macrophages were then primed with the medium supplemented with 20 ng/ml of IFNγ and 1 mg/ml of LPS (Sigma-Aldrich) to be differentiated into M1 phenotype or with 20 ng/ml of IL-4 to be differentiated into M2 phenotype. The incubation time was 6 h and 24 h, respectively, under the two stimulating conditions.

### Co-cultures of cancer cells and macrophages

Panc1 and BxPC-3 cells (2 × 10^5^ cells) were seeded onto 6-well plates. The cells were co-cultured with M1 or M2-polarized macrophages over a distance using a transwell system (BD Falcon™; Becton Dickinson, Franklin Lakes, NJ, USA) for 48 h. Cancer cells were then trypsinized and reseeded on a 96-well plate at 1000 cells per well. The cells were treated with chemotherapeutic agents for 48 h, and cell viability for quantitative evaluation was determined using an XTT Cell Proliferation Kit II (Roche Life Science, Indianapolis, IN, USA) according to the manufacturer’s protocol. After the treatment with chemotherapeutic agents, dose-response curves were drawn, and the IC50 values were determined by GraphPad Prism 8 version 8.00 (GraphPad Software, Inc., La Jolla, CA, USA).

### Western blotting analysis

Cells were washed with cold phosphate-buffered saline (PBS) and lysed with the SDS buffer. Whole-cell lysates were loaded into each lane of an 8% SDS-polyacrylamide gel and electrophoretically transferred to polyvinylidene difluoride membranes (Hybond-P; GE Health Care, Buckinghamshire, UK). Membranes were incubated with primary antibodies against CD80 (EPR1157; Abcam, Cambridgeshire, UK), CD68 (EPR1392Y; Abcam), CD204 (MSR-A; TransGenic), E-cadherin (Cell Signaling Technology, Danvers, MA, USA), vimentin (Cell Signaling Technology), α-SMA (Abcam), and β-actin (Sigma-Aldrich) overnight at 4 °C. The secondary antibodies used were horseradish peroxidase-conjugated antibodies against rabbit IgG or mouse IgG (GE Healthcare). The blots were visualized using an Amersham ECL chemiluminescence system (GE Healthcare) according to the manufacturer’s protocol.

### Cell migration and invasion assays

In vitro migration and invasion assays were examined in the chambers of 8-μm transwell inserts with or without Matrigel (BD Falcon™), respectively. Cancer cells were incubated in serum-free medium at the top chamber of each well insert, and macrophages were incubated in the lower chamber for 48 h at 37 °C. Cells in the top chamber were fixed with 4% paraformaldehyde and stained with 0.5% crystal violet. The stained cells were counted under a light microscope at a magnification of × 200.

### Mouse xenograft model

Athymic female BALB/c nu/nu nude mice aged 4–6 weeks were purchased from CLEA Japan (Tokyo, Japan). The animal care and experimental procedures were conducted in accordance with the regulations of the Animal Care and Use Committee of Okayama University. To compare the effects of co-inoculation of BxPC-3-luc cells and M2 macrophages in a subcutaneous tumor model, 2 × 10^6^ BxPC-3-luc cells in 100 μl of PBS with or without 2 × 10^6^ M2 macrophages were injected subcutaneously into the dorsal flank of the mouse. In the peritoneal dissemination model, 5 × 10^6^ BxPC-3-luc cells with or without 1 × 10^7^ M2 macrophages in 500 μl of PBS were injected into the peritoneal cavity. The numbers of injected tumor cells were empirically determined based on our previous preliminary experiments. The ratio of co-injected macrophages to tumor cells was based on the previous report [15]. Gemcitabine was administered intraperitoneally at a dose of 200 μg on the 16th, 23rd, and 30th days. Tumor burden was quantified after intraperitoneal injection of VivoGlo™ Luciferin (Promega, Madison, WI, USA) and imaged with an IVIS Spectrum system (Caliper Life Sciences, Waltham, MA, USA).

### Immunohistochemistry

Formalin-fixed tumors were embedded in paraffin, sectioned, and stained with hematoxylin and eosin. For M2 macrophage localization and EMT induction, sections were immunostained with anti-CD204 (MSR-A; TransGenic) and anti-vimentin (Cell Signaling Technology) antibodies, respectively.

### Statistical analysis

Data are shown as means ± standard deviation (SD). For comparisons between two groups, significant differences were determined using Student’s *t*-test. *P* values < 0.05 were considered significant. Statistical analysis for overall survival was performed using the Kaplan-Meier method with the log-rank test. The analysis for IC50 was performed using GraphPad Prism 8 (GraphPad Software, Inc).

## Results

Tumor-associated macrophages in the cytology-positive peritoneal microenvironment in pancreatic cancer.

**Fig. 1:**
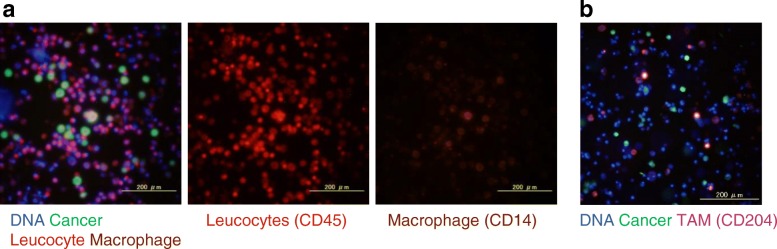
Immunofluorescence assays of cells comprising the peritoneal microenvironment. **a**. Clinical samples of peritoneal washes obtained from a cytology-positive case. After TelomeScan was infected at an MOI of 1 for 24 h, and cancer cells were identified as GFP-positive cells, leukocytes and monocytes were stained with A647-labeled anti-CD45 antibodies and PE-labeled anti-CD14 antibodies, respectively. **b**. Clinical samples of peritoneal wash obtained from another cytology-positive case were analyzed. GFP-positive cells are detected after TelomeScan. The polarity of macrophages to M2 phenotype is confirmed with PE-conjugated anti-CD204

To first investigate the peritoneal cancer microenvironment, peritoneal lavage fluid was obtained from 34 pancreatic cancer patients, and the cellular component was analyzed. Since it is difficult to discriminate cancer cells from surrounding normal cells in cytological samples, a virus-based assay was used. This assay used a cancer-imaging virus, TelomeScan, that expresses GFP in a telomerase activity-dependent manner [13, 14]. Of the 34 clinical samples, 5 were positive on cytology and subjected to imaging analysis. In combination with immunofluorescence staining, GFP-positive cancer cells were observed among numerous co-existing CD45-positive leukocytes (Fig. 1a). Further analysis showed that these CD45-positive cells contained CD14-positive macrophages (Fig. 1a). Macrophages are known to polarize to either M1 type or M2 type depending on their environments. Immunostaining of the cells from other peritoneal lavage fluid demonstrated that they were predominantly CD204-positive M2-type macrophages (Fig. 1b). Further image discrimination between M1- and M2-type macrophages using the other cellular markers including CD80 (M1 marker) was not successful; nevertheless, these observations suggested that macrophages were relatively skewed towards M2 in the peritoneal cavity with positive cytology, and pancreatic cancer cells exfoliated from a primary lesion would encounter such macrophages as tumor-associated macrophages (TAMs) in the environment of the peritoneal cavity.

### TAMs interact with pancreatic cancer cells to affect their phenotype

**Fig. 2:**
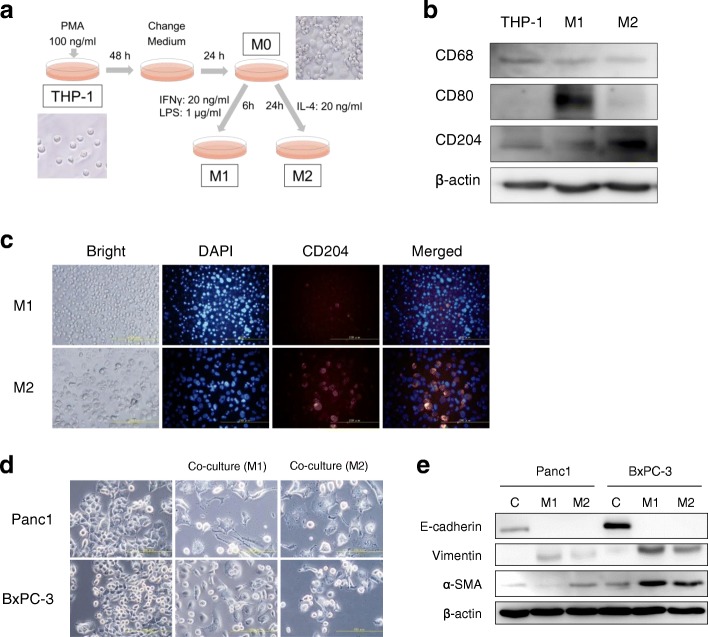
Induction of the EMT in cancer cells. **a**. Induction process in THP-1 cells to M1 or M2 macrophages. **b**. Western blot analyses of CD80, an M1 macrophage marker, CD204, an M2 macrophage marker, and CD68, a pan-macrophage marker. **c**. Immunofluorescence staining of M1 and M2 type macrophages. **d**. Morphological changes after Panc1 and BxPC-3 cells were co-cultured with macrophages. **e**. Western blot analyses show that Panc1 and BxPC-3 cells co-cultured with macrophages express vimentin and α-SMA proteins but have decreased E-cadherin

To explore the potential interactions between pancreatic cancer cells and TAMs, THP-1 monocytic cells were artificially manipulated into macrophages and further polarized to M1 or M2 types. The polarized phenotype was then analyzed by Western blotting in which CD68, CD80, and CD204 were used as markers of macrophages and of polarization to M1 or M2 phenotype. THP-1 cells were successfully polarized to either M1- or M2-macrophages, as shown by up-regulated CD80 or CD204 protein expression on Western blotting (Fig. 2b), respectively. The immunofluorescent staining demonstrated that M2-polarized macrophages expressed CD204 more prominently than M1-polarized ones (Fig. 2c).

The next step was to examine whether pancreatic cancer cells interact with TAMs in the peritoneal cavity. Mimicking that situation, the pancreatic cancer cells and THP-1-derived macrophages were co-cultured over a distance, and this resulted in the morphological change of pancreatic cancer cells to spindle shapes (Fig. 2d). Whether the induced morphological change of pancreatic cancer cells was related to the epithelial-to-mesenchymal transition (EMT) was then examined. Panc1 and BxPC-3 cells co-cultured with M2-polarized macrophages decreased their expression of E-cadherin, and the BxPC-3 and Panc1 cells increased their expressions of either one or both of vimentin and α-SMA in (Fig. 2e). Pancreatic cancer cells co-cultured with M1-macrophages also showed characteristic EMT changes similar to or somehow more prominent than those co-cultured with M2-macrophages. The results demonstrated that pancreatic cancer cells can be affected by TAMs even in the situation of indirect co-culture and irrespective of macrophage polarization status, which induces the EMT-phenotype in pancreatic cancer cells.

### The EMT activates cell motility and decreases sensitivity to chemotherapeutic agents in pancreatic cancer cells

**Fig. 3:**
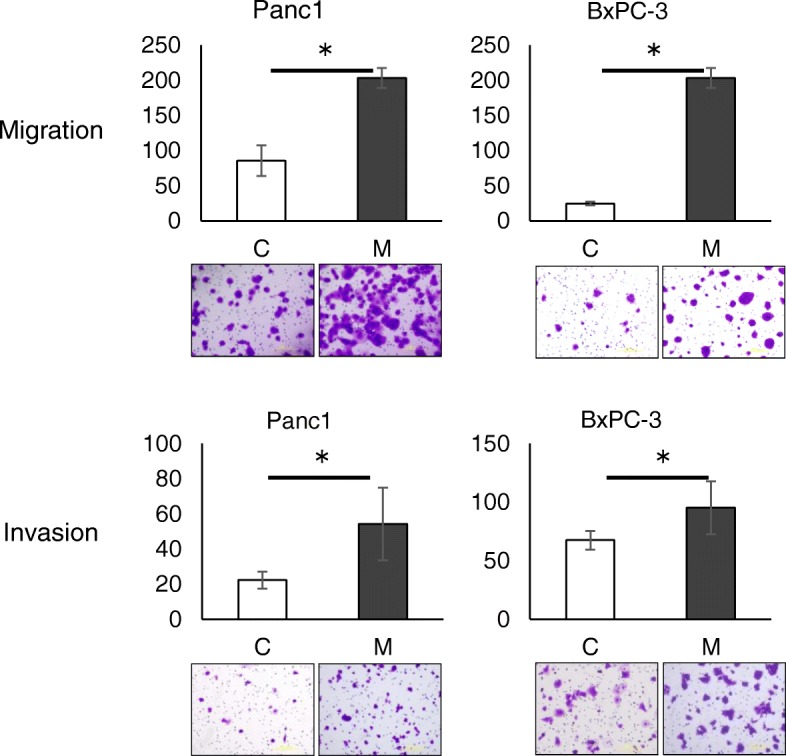
Tumor-associated macrophages promote migration and invasion. After co-culture with macrophages, Panc1 and BxPC-3 cells have enhanced migration and invasion abilities. C: control. M: co-culture with macrophages. Results are from representative experiments in quadruplicate and are shown as means ± S.D. * *p* < 0.01

To further investigate whether the EMT induced by TAMs actually affected the malignant phenotype of the pancreatic cells, migration and invasion ability were then compared between Panc-1 cells co-cultured with M2 macrophages and the parental ones. Co-culture with M2 macrophages increased the numbers of migrating and invading cells over those of the parental cells. This transition was also confirmed in BxPC-3 cells (Fig. 3). These results demonstrated that co-existing macrophages induced the EMT in pancreatic cancer cells, which made them more migratory and invasive.

**Fig. 4:**
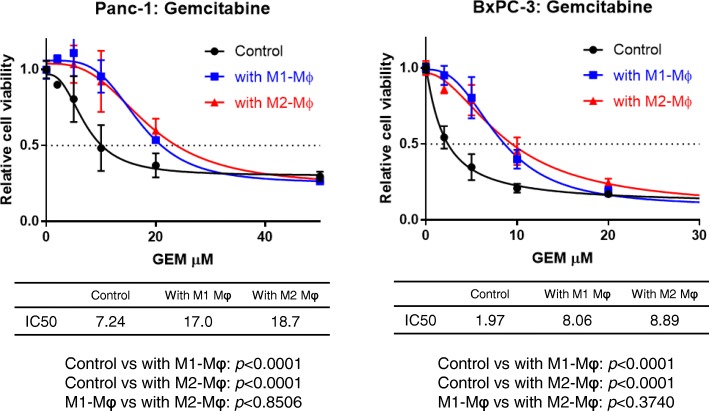
Cancer cells show chemoresistance after co-culture with macrophages. Panc1 and BxPC-3 cells cultured with macrophages were exposed to gemcitabine for 48 h at different concentrations. Cell viability was measured in triplicate by XTT assay, and representative results are shown as means ± S.D. The calculated IC50 is shown in the lower tables

Next, whether co-existing macrophages would affect the sensitivity of pancreatic cancer cells to the representative chemotherapeutic agents was examined. After Panc1 and BxPC-3 cells co-cultured with M1 or M2-polarized macrophages were treated with gemcitabine, both cells developed more resistance to the test agents (Fig. 4). These effects seemed to be almost equal for M1 and M2 macrophages, which corresponds to the equal effect of M1 and M2 macrophages in EMT induction. In the case of 5-fluorouracil (5-FU), only Panc1 cells co-cultured with M2-polarized macrophages developed more resistance to 5-FU, while BxPC-3 cells were so sensitive to 5-FU by nature that neither M1 nor M2 macrophages affected it (Additional file 1).

These results demonstrated that the EMT phenotype induced by macrophages conferred onto pancreatic cells a more malignant phenotype and refractoriness. In addition, these observations suggested the potential interaction between pancreatic cancer cells and TAMs via indirect contact in the peritoneal cavity.

### Accelerated growth of subcutaneous or peritoneal tumors by TAMs

**Fig. 5:**
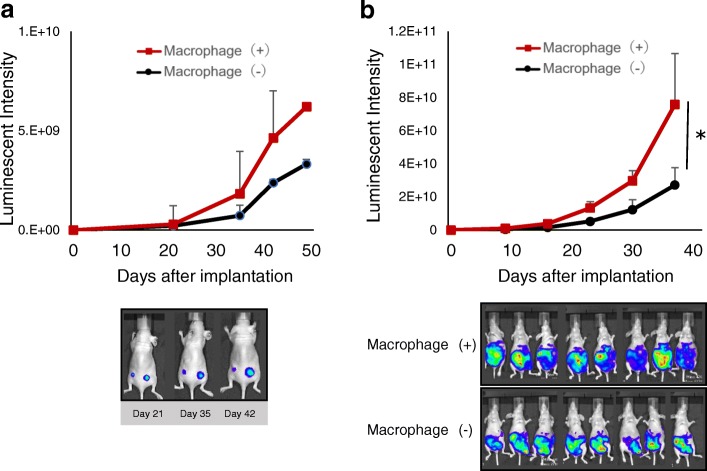
Tumor-associated macrophages promote tumorigenesis. **a** Subcutaneous BxPC-3-luc tumors co-inoculated with macrophages tend to grow more rapidly than controls. *N* = 3/each tumor. Pictures: Representative IVIS imaging on days 21, 35, and 42. **b** Peritoneal BxPC-3-luc tumors co-inoculated with macrophages grow significantly more rapidly than controls. *N* = 8/treatment group. * *p* < 0.01. Pictures: Representative IVIS imaging on day 37

The effects of TAMs on pancreatic cancer cells were also examined in vivo. When cells were subcutaneously inoculated with or without M2 macrophages, tumors with co-injection of M2 macrophages grew more rapidly than tumors alone (Fig. 5a). Immunostaining tumor confirmed that co-injected positive macrophages around cancer cells continued to express CD204. Furthermore, in such tumors, cancer cells were positive for vimentin, suggesting that those cancer cells underwent the EMT in vivo (Additional file 2). The effect of macrophages in the peritoneal dissemination model was further examined. As in the subcutaneous tumor model, pancreatic cancer cells in the peritoneal cavity grew more rapidly than tumors without co-injection of M2 macrophages (Fig. 5b). These rapidly growing peritoneal tumors killed mice significantly faster (Additional file 3a), while the M2-polarized macrophages themselves were harmless (Additional file 3b).

### Tumors with TAMs developed chemoresistance in vivo

**Fig. 6:**
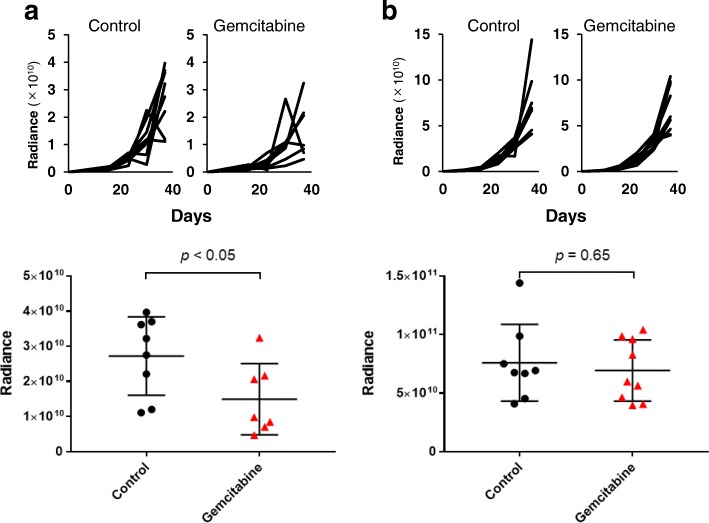
Co-inoculated macrophages make xenografted pancreatic cancer BxPC-3-luc more resistant to chemotherapy than control. Mice intraperitoneally xenografted with BxPC-3-luc cells were treated with gemcitabine 3 times weekly. Growth curves are shown in the upper panels, and tumor burdens on day 37 are plotted in the lower panels where bars indicate the means and 95% confidence intervals. While chemotherapy significantly suppresses the growth of peritoneal tumors (**a**), co-inoculation of macrophages offsets the therapeutic effect (**b**). * *p* < 0.05 (unpaired *t*-test)

Finally, whether macrophages in the peritoneal cavity affected sensitivity to the chemotherapeutic agents in peritoneally disseminated pancreatic cancer was examined. As observed in vitro, co-injected macrophages rendered pancreatic cancer more chemo-resistant than tumors without co-injection of M2 macrophages in the peritoneal dissemination model (Fig. 6).

## Discussion

In clinical peritoneal washes with positive cytology, macrophages skewed towards M2 were always present in the ipTME. In this study, thus, the interaction between macrophages and pancreatic cancer was investigated to explore the underlying mechanism of the development and promotion of peritoneal dissemination. These macrophages could interact with pancreatic cancer cells to render them more migratory and invasive through induction of the EMT and thereby promote growth and chemoresistance. Therefore, TAMs in the peritoneal cavity of pancreatic cancer patients have a potential role in peritoneal dissemination as a component of the ipTME.

The TME is now recognized as a critical factor fostering cancer progression, and macrophages are known to be major components of the TME [16]. In fact, it has been reported that TAMs promote tumor progression in many types of cancers [17–20]. The clinical correlation between the amount of TAMs in solid tumor and the prognosis of various cancers has been suggested [21–25]. Although peritoneal dissemination is the most frequent mode of metastasis of pancreatic cancer, the roles of TAMs in the peritoneal environment were unclear.

Immunostaining of the intraperitoneal microenvironment showed that many macrophages existed together with pancreatic cancer cells (Fig. 1), and they were polarized to M2. It is still unclear whether intraperitoneal macrophages are always abundant and polarized to M2 phenotype. Yamaguchi et al. recently demonstrated that TAMs were present around gastric cancer cells invading the peritoneum, and the TAMs became more abundant as gastric cancer progressed [17]. On the other hand**,** macrophages function with an M1-like phenotype during the initiation of tumor and switch to an M2-like phenotype when the tumor begins to invade and metastasize [26, 27]. Therefore, the phenotype of TAM may vary during different stages of tumor progression [28, 29], and TAMs generally tend to exhibit an M2-like phenotype at later stages of cancer progression. Although which factors in the peritoneal cavity polarize macrophages remains unclear, the existence of cancer cells would possibly make them polarized, or the environment in the peritoneal cavity itself might render them to be polarized to M2.

The influence of M1- or M2-polarized macrophages on pancreatic cancer cells was demonstrated. Indirect co-culture with macrophages induced the EMT in pancreatic cancer cells, irrespective of the polarization status of macrophages (Fig. 2e), but the mechanism of TAMs inducing the EMT of pancreatic cancer cells was not elucidated in this study. Recent reports by others suggested that IL-8 [30], CCL20 [31], and the TLR4/IL10 signaling pathway [15] might be associated with EMT-induction in an in vitro culture system, and further investigations will be necessary to uncover the precise mechanism of EMT induction through the interplay between pancreatic cancer and TAMs.

Pancreatic cancer cells in which the EMT was induced by macrophages showed greater ability to migrate and invade than control cells (Fig. 3). Even in vivo, co-inoculation of pancreatic cancer cells with M2 macrophages resulted in larger tumors than cancer cells alone (Fig. 5a) in a subcutaneous model, as well as a peritoneal dissemination model (Fig. 5b). Although the effect on malignant potential was demonstrated only by co-culture or co-inoculation with M2 macrophages, M1 macrophages might also have a similar potential based on the results of comparable EMT induction. Considering the reports by others suggesting that TAMs induce the EMT in cancer cells as part of the TME [15, 25, 32], as well as the evidence that the EMT program appears to be critical to migration and invasion in most cancers [33], TAMs would have substantial impact on the malignant potential of pancreatic cancer cells even in the peritoneal cavity. Furthermore, co-culturing with macrophages rendered pancreatic cancer cells more resistant to gemcitabine and 5-FU (Fig. 4, Additional file 1), suggesting that the EMT induced by macrophages conferred chemoresistance. Emerging evidence has shown that the EMT is associated with chemoresistance [34–37], although the possible mechanism of inducing chemoresistance varies for different chemotherapeutic agents. In the present study, changes of expressions of anti-apoptotic proteins such as bcl-2, mcl-1, and transporter proteins were examined in pancreatic cancer cells co-cultured with TAMs, but no significant changes were observed (data not shown). Therefore, an unproven mechanism may underlie TAM-induced chemoresistance. Interestingly, not only M2 type macrophages, but M1 type macrophages also induced the EMT and chemoresistance in cancer cells (Fig. 2e, Fig. 4, Additional file 1). The possibility that M1 and M2 types can be interchangeable or M1 phenotype might have been switched to M2 type during co-culture has not been excluded [38]. Intriguingly, accumulating evidence shows that TAMs often share phenotypes of both M1 and M2 rather than being divided into the two types [39, 40]. Nevertheless, cancer cells treated with macrophages were more chemoresistant than parental cells, suggesting that TAMs might have the potential to confer refractoriness in cancer cells.

Based on the present results, TAM would be an attractive therapeutic target even in peritoneal dissemination of pancreatic cancer. Expectations for TAM-targeting therapy have provoked many strategies, including blocking the CSF1 receptor signal pathway by neutralizing antibody and small molecule inhibitors [41–43], and direct killing of macrophages by trabectedin [44] or zoledronic acid [45], and various clinical trials focused on macrophages are actually under way [46, 47]. Targeting TAM would alter malignant phenotypes and chemoresistance, and therefore synergistic effects with chemotherapy would also be expected.

There were some limitations in this study. First, although in vitro experiments were carried out under indirect co-culture of cancer cells only with macrophages, the intraperitoneal microenvironment is composed of various kinds of cells other than macrophages. Therefore, a diverse and complex interplay must exist, and this study cannot completely reproduce it. In addition, the direct contact with macrophages may have some effect on cancer cells, which was not investigated in the present study. Second, the EMT was proposed as a possible reason for chemoresistance, but its concrete mechanism is unknown. In addition, to generalize the effects of macrophages on chemo-refractoriness of peritoneal dissemination, it is desirable to investigate it with other chemo-agents that act by different mechanisms. Thus, the details of the regulation of chemo-sensitivity by the EMT and macrophages need to be further addressed.

## Conclusion

The EMT induced by TAMs could be a possible mechanism fostering peritoneal dissemination and causing chemoresistance in pancreatic cancer. TAMs would be a novel therapeutic target for overcoming chemoresistance and improving treatment outcomes in cases of peritoneal dissemination in this dismal disease.

## Additional files

Additional file 1: Tumor-associated macrophages induce chemoresistance. XTT assays show that chemoresistance is induced in Panc1cells by co-culture with macrophages, but not in BxPC-3 cells. (PDF 123 kb)

Additional file 2: Immunohistochemistry of subcutaneous BxPC-3-luc tumors co-injected with or without M2 macrophages. CD204 indicates M2-polarized macrophages, and vimentin indicates cells that underwent the EMT. Immunostaining of a tumor demonstrates expression of vimentin along with CD204 only in the tumor co-injected with M2-polarized macrophages, suggesting that macrophages induce the EMT in vivo (PDF 253 kb)

Additional file 3: (a) Kaplan-Meier analysis of survival in tumor-bearing mice co-inoculated with or without macrophages. *N* = 8/group. Mice with peritoneal dissemination with macrophages show shorter periods of survival than those without macrophages. * *p* < 0.05. (b) Kaplan-Meier analysis of survival in tumor-free mice intraperitoneally inoculated only with M2-polarized macrophages. *N* = 5. (PDF 116 kb)

## Abbreviations

EMT: Epithelial-to-mesenchymal transition
FBS: Fetal bovine serum
GFP: Green fluorescent protein
hTERT: Human telomerase reverse transcriptase
LPS: Lipopolysaccharide
TAM: Tumor-associated macrophage
