# Supplementary material for: The epithelial-to-mesenchymal transition induced by tumor-associated macrophages confers chemoresistance in peritoneally disseminated pancreatic cancer
Source: J Exp Clin Cancer Res. 2018 Dec 11;37:307. doi: 10.1186/s13046-018-0981-2 (PMC6288926; doi:10.1186/s13046-018-0981-2)
Supplement: Supplementary file 1 — Tumor-associated macrophages induce chemoresistance. XTT assays show that chemoresistance is induced in Panc1cells by co-culture with macrophages, but not in BxPC-3 cells. (PDF 123 kb) [file 13046_2018_981_MOESM1_ESM.pdf]

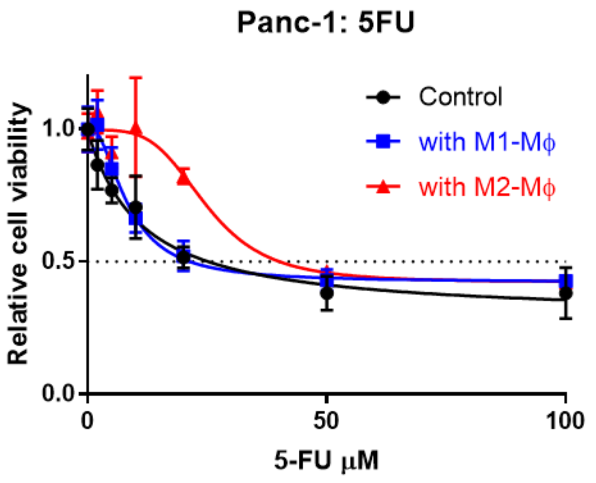

	Control	With M1 Mφ	With M2 Mφ
IC50	11.1	8.3	25.0

Control vs with M1-Mφ: $p<0.1288$
Control vs with M2-Mφ: $p<0.0001$
M1-Mφ vs with M2-Mφ: $p<0.0001$

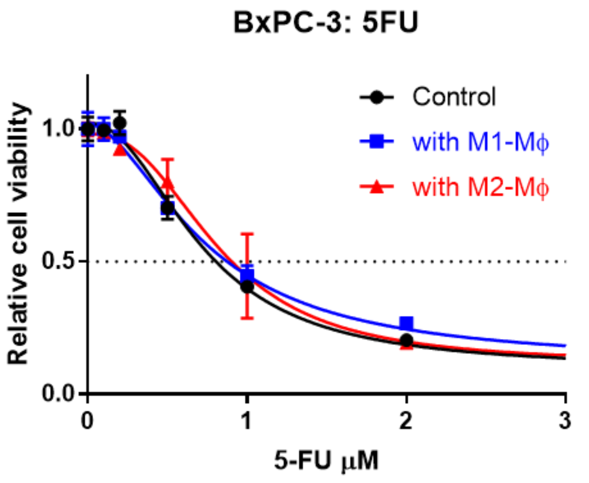

	Control	With M1 Mφ	With M2 Mφ
IC50	0.71	0.74	0.83

Control vs with M1-Mφ: $p<0.1120$
Control vs with M2-Mφ: $p<0.3283$
M1-Mφ vs with M2-Mφ: $p<0.2773$
