# Supplementary figures and images for: The epithelial-to-mesenchymal transition induced by tumor-associated macrophages confers chemoresistance in peritoneally disseminated pancreatic cancer
Source: J Exp Clin Cancer Res. 2018 Dec 11;37:307. doi: 10.1186/s13046-018-0981-2 (PMC6288926; doi:10.1186/s13046-018-0981-2)

H.E. (× 100)

CD204 (× 400)

Vimentin (× 400)

TAMs (-)

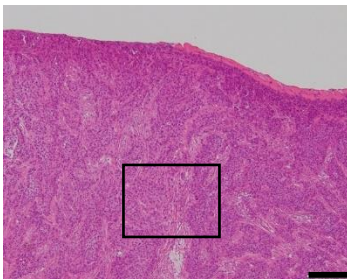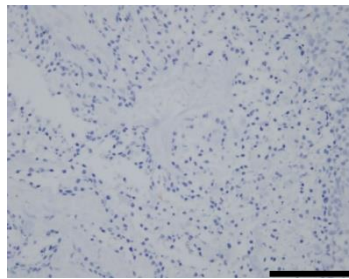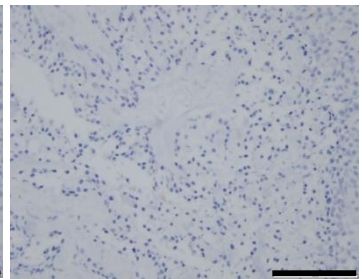

TAMs (+)

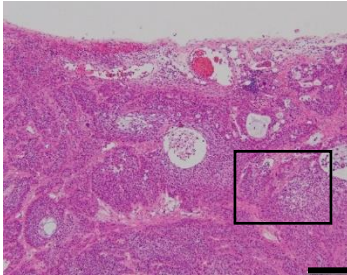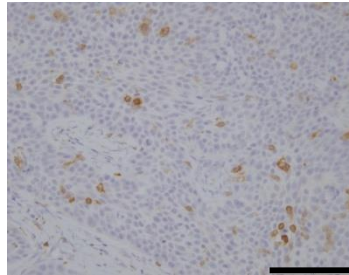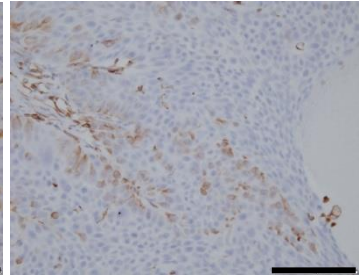

scale bar : 200  $\mu$ m

Supplement: Supplementary file 2 — Immunohistochemistry of subcutaneous BxPC-3-luc tumors co-injected with or without M2 macrophages. CD204 indicates M2-polarized macrophages, and vimentin indicates cells that underwent the EMT. Immunostaining of a tumor demonstrates expression of vimentin along with CD204 only in the tumor co-injected with M2-polarized macrophages, suggesting that macrophages induce the EMT in vivo (PDF 253 kb) [file 13046_2018_981_MOESM2_ESM.pdf]

a

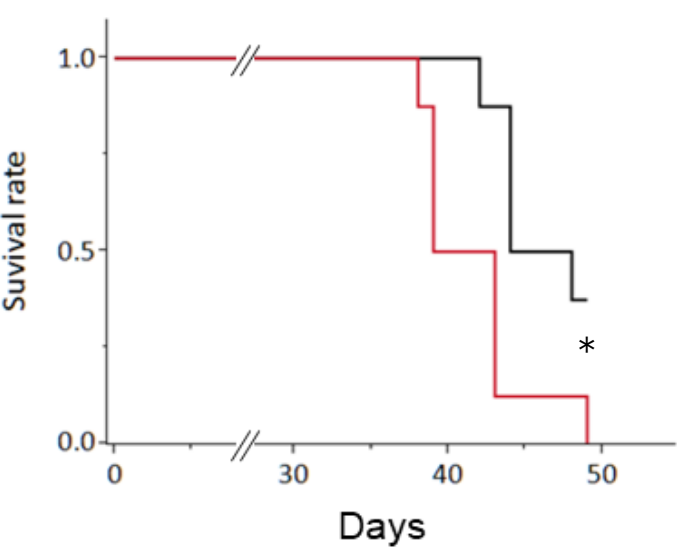

b

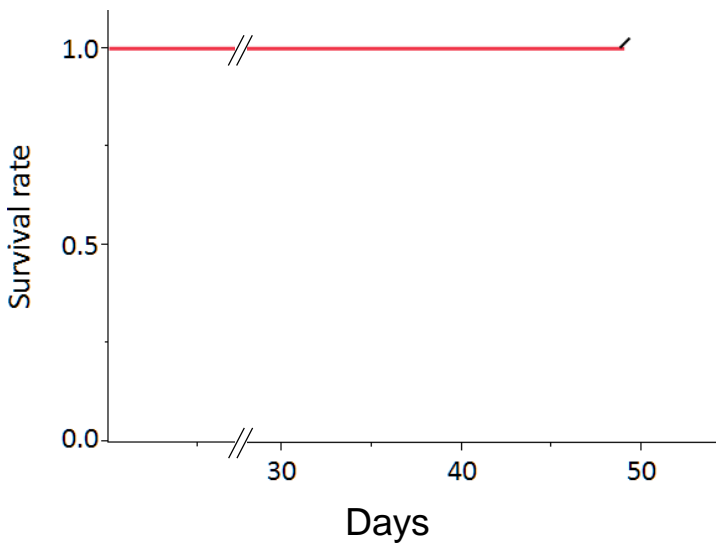

Supplement: Supplementary file 3 — (a) Kaplan-Meier analysis of survival in tumor-bearing mice co-inoculated with or without macrophages. N = 8/group. Mice with peritoneal dissemination with macrophages show shorter periods of survival than those without macrophages. * p < 0.05. (b) Kaplan-Meier analysis of survival in tumor-free mice intraperitoneally inoculated only with M2-polarized macrophages. N = 5. (PDF 116 kb) [file 13046_2018_981_MOESM3_ESM.pdf]
